# Bone Marrow Transfer in Relapsing-Remitting EAE Ameliorates Disease at First Remission, with No Synergistic Effect upon Co-Transplantation with Mesenchymal Stem Cells

**DOI:** 10.3390/vaccines9070736

**Published:** 2021-07-03

**Authors:** Giovanni Ferrara, Federico Ivaldi, Gianluigi Mancardi, Nicole Kerlero de Rosbo, Antonio Uccelli

**Affiliations:** 1 Ospedale Policlinico San Martino IRCCS, 16132 Genoa, Italy; ivaldi@email.it (F.I.); auccelli@neurologia.unige.it (A.U.); 2 Department of Neurology, Rehabilitation, Ophthalmology, Genetics, Maternal and Child Health, University of Genoa, 16132 Genoa, Italy; glmancardi@neurologia.unige.it (G.M.); nicole.kerleroderosbo@unige.it (N.K.d.R.); 3 IRCCS, ICS Maugeri, 27100 Pavia, Italy

**Keywords:** stem cell therapy, multiple sclerosis, intensive immunosuppression, experimental autoimmune encephalomyelitis, bone-marrow cells, mesenchymal stem cells

## Abstract

Multiple sclerosis (MS) is a neurological disorder characterized by an autoimmune response, demyelinating plaques and axonal damage. Intense immunosuppression (II) followed by autologous hematopoietic stem cell transplantation has been proposed as a treatment in severe forms of MS. We have used murine relapsing-remitting (RR) experimental autoimmune encephalomyelitis (RR-EAE) to evaluate the transplantation of syngeneic bone marrow cells (BMC) after II, in combination with mesenchymal stem cells (MSCs) as a new therapeutic adjunct capable of improving immune reconstitution. In EAE-affected mice treated with BMC alone, we observed a drastic reduction in the clinical course only during the early RR phase of the disease. There was no difference in the RR-EAE clinical course between mice treated with BMC alone and co-transplanted mice. To analyze the immune reconstitution, we quantified the circulating immune cells in naïve and RR-EAE-affected mice after II, with BMC alone or in combination with MSC. Although II resulted in reduced numbers of circulating immune cells, reconstitution did not differ in co-transplanted mice. During the early phase of the disease, IL-4 was significantly elevated in co-transplanted mice, as compared to those treated with BMC alone. These data suggest that BMC transplantation after II transiently ameliorates the clinical symptoms of RR-EAE, but that co-transplantation with MSC has no synergistic effect.

## 1. Introduction

Multiple sclerosis (MS) is an autoimmune disorder driven by autoreactive T-cell and B cells, resulting in focal inflammatory infiltrates in the central nervous system (CNS), demyelinating areas and axonal loss [1,2]. It is generally believed that both genetic and environmental factors contribute to the development and progression of the disease. The peripheral autoimmune response plays a critical role in the development of the disease, especially during its early phases [2] and is the target of the currently available disease modifying treatments (DMTs). In aggressive forms of MS that are not responsive to currently available DMTs, intense immunosuppression, followed by the infusion of autologous hematopoietic stem cells (HSCs), is highly effective in stopping disease activity and progression [3]. The widespread utilization of this procedure is limited by the peritransplant mortality, associated with infectious complications in the early aplastic phase, which have decreased, however, in recent years to less than 1% [4]. Experimental autoimmune encephalomyelitis (EAE) is the purported animal model for MS, which is induced by T-cell and B-cell autoreactivity to myelin components [5]. Depending on the mouse strain and myelin antigen used, murine EAE can present with different clinical courses, including relapsing-remitting EAE (RR-EAE) induced in SJL/J mice with proteolipid protein (PLP) peptides 139–151 [5,6]. Evidence from preclinical studies suggests that the infusion of HSC-containing bone marrow cells (BMC) in intensely immunosuppressed mice, or of bone marrow-expanded mesenchymal stem cells (MSCs) could ameliorate the clinical course, decreasing demyelination, inflammatory infiltrates and axonal loss, and promoting remyelination in EAE [7,8,9,10]. It has been demonstrated that this procedure suppresses the autoimmune response in animal models and induces tolerance to the immunizing antigen; thus, chronic-relapsing EAE could be prevented successfully through immunosuppression with cyclophosphamide, followed by syngeneic bone marrow transplantation [11]. Treatment with MSCs derived from the bone marrow ameliorates EAE [7,12], exerting a remarkable immune-modulating activity and tissue repair, mainly due to bystander mechanisms. A very important biological effect of MSCs, which is of particular relevance for BMC transplantation, is their capacity to promote hematopoietic engraftment. This effect is likely to be related to the close interaction between these two types of cells within the bone marrow [13]. In fact, studies have reported that co-transplantation of MSCs and BMC accelerates lymphocyte recovery after immunosuppression [14], reducing the risk of graft failure and inducing a rapid hematopoietic recovery [15]. On the basis of the above data, we hypothesized that co-transplantation of BMC with MSCs following intense immunosuppression could synergize the therapeutic effects of both treatments in EAE-affected mice to facilitate the reconstitution of a tolerant new immune system with a consequent reduction in disease severity. In this study, we investigated the effect of a BMC transfer after intense immunosuppression in RR-EAE and found that it ameliorated the clinical symptoms of EAE during the early stage of the disease. However, co-transplantation with MSCs did not significantly improve lymphocyte recovery and did not contribute to a synergistic effect with BMC administration in RR-EAE pathogenesis and progression, despite some evidence of anti-inflammatory effects, with elevated levels of the anti-inflammatory cytokine IL-4 in the plasma of co-transplanted mice.

## 2. Materials and Methods

### 2.1. RR-EAE Induction

SJL/JCrl female mice were purchased from Charles River (Calco, Italy). All mice were housed in pathogen-free conditions with access to food and water ad libitum. RR-EAE was induced in female wild type mice (7 weeks of age, weighing 18.5 ± 1.5 g) via a subcutaneous injection at two different sites in the right and left flanks with an emulsion (200 μL total) containing 200 μg of proteolipid protein peptides spanning amino acids 139–151 (PLP) (Espikem, Florence, Italy) in incomplete Freund’s adjuvant (Sigma-Aldrich, Merck KGaA, Darmstadt, Germany) supplemented with 600 μg Mycobacterium tuberculosis (strain H37RA; Difco). Mice were injected in the tail vein with 400 ng pertussis toxin (List Biological, Campbell, CA, USA) in 100 μL of phosphate buffer saline solution (PBS, pH 7.6) immediately and 48 h after the immunization. The mice were scored daily for clinical manifestations of RR-EAE by a single observer and graded as listed here: 0, clinically normal; 1, decreased tail tone or weak tail only; 2, hind limb weakness (paraparesis); 3, hind limb paralysis (paraplegia); 4, weakness of front limbs with paraparesis, paraplegia (quadriparesis); 5, death [12]. All applicable international, national, and/or institutional guidelines for the care and use of animals were followed (Decreto Legislativo 4 marzo 2014, n. 26, legislative transposition of Directive 2010/63/EU of the European Parliament and of the Council of 22 September 2010 on the protection of animals used for scientific purposes). The research protocol was approved by the Ethical Committee for Animal Experimentation of the IRCCS Ospedale Policlinico San Martino, Genoa, Italy, and by the Italian Ministry of Health (project No. 917/2016-PR).

### 2.2. Generation of Bone Marrow-Derived MSCs

Primary bone marrow-derived MSCs were obtained as described previously [7]. Briefly, bone-marrow cells were flushed from the femur and tibia of SJL mice and passed through a 70-μm nylon cell strainer (Becton–Dickinson, Franklin Lakes, NJ, USA). The cell suspension was seeded in the presence of Murine Mesencult as medium (Stem Cell Technologies, Cambridge, UK) and MSCs were expanded and tested after 8 passages. They were identified as MSCs according to their expression of CD9, SCA-1 and CD44 and their lack of expression of CD45 and MHC II markers, assessed using flow cytometry with appropriate conjugated monoclonal antibodies: FITC anti-CD9, PE anti-SCA1, APC anti-CD44, PerCp anti-CD45 and PB anti-MHC II (Biolegend, San Diego, CA, USA). MSCs were also tested for their ability to reduce T-cell proliferation [7].

### 2.3. Intense Immunosuppression and Cell Therapy

Total-body irradiation (TBI) of mice was carried out with a RADGIL 2 (Gilardoni, Lecco, Italy) linear accelerator to induce intense immunosuppression. The EAE-affected mice were irradiated at disease onset (11 days post-immunization) with a lethal dose (8 Gy) of γ radiation and either immediately transplanted with whole bone marrow cells (BMC), (10 × 10^6^ per mouse in 100 μL PBS injected in the tail vein) freshly isolated from femurs of syngeneic 8-week-old donor female mice (EAE BMC group), or co-transplanted with whole BMC (as above) together with 1 × 10^6^ bone-marrow derived MSCs per mouse (EAE BMC MSC group). Control EAE-affected mice were not irradiated and they received only the same volume of PBS (EAE group).

### 2.4. Flow Cytometry

Blood (50 μL) for immune cell counting was obtained by means of retro-orbital collection and immediately transferred into 1.5-mL EDTA-coated tubes (Sarstedt, Nümbrecht, Germany). Blood samples were treated with ammonium–chloride–potassium lysing buffer (50 μL) for 5 min at 4 °C to lyse red blood cells. Samples were resuspended in 100 μL of flow cytometry buffer (PBS, pH 7.2, containing 0.5% bovine plasma albumin) and surface-marker staining of immune cells was performed, using the appropriate conjugated monoclonal antibodies (1:1000 PerCp anti-CD45 pan-immune-cell marker; APC anti-CD3 for T cells, PE anti-CD19 for B cells, and FITC anti-CD11b for monocytes/macrophages cells, all from Biolegend) for 30 min at RT. CountBright™ (Thermofisher, Waltham, MA, USA) absolute counting beads mixed with the cell sample were used according to the manufacturer’s instructions to calculate the absolute number of the various types of immune cells in the sample.

### 2.5. ELISA

Cytokines were evaluated in plasma from RR-EAE-affected mice using ELISA Standard Set kits (IL-4 and IFN-γ, ELISA MAX, Biolegend), as per the manufacturer’s instructions. Briefly, 96-well ELISA plates coated at 4 °C overnight with the relevant capture antibodies were washed with PBS supplemented with 0.1% Tween 20 and blocked with PBS containing 5% fetal bovine serum for 2 h at room temperature. The test plasma (50 μL) was added and the plates were incubated for 2 h at room temperature. Plates were washed and incubated with the relative horseradish peroxidase-coupled detection antibodies for 1 h at room temperature. The plates were washed, substrate (3,3′,5,5′tetramethylbenzidine, Sigma-Aldrich) was added, and the plates were developed for 20–30 min. The reaction was stopped with H_2_SO_4_ (2N) and plates were read at OD 450 nm on a Multilabel Victor3 reader (Perkin Elmer, Waltham, MA, USA).

### 2.6. Statistical Analysis

The results were analyzed with GraphPad Prism software (version 6.01; GraphPad Software, San Diego, CA, USA). The animals had been randomly assigned to groups. The analyzed data passed a normality test (Shapiro–Wilk normality test) and intergroup differences were analyzed by means of one-way ANOVA, followed by Tukey’s multiple comparisons post hoc tests. For comparisons of two sets of data, we used the Mann–Whitney test. Differences between groups were considered to be significant at *p* ≤ 0.05.

## 3. Results

### 3.1. Immunosuppressed, BMC-Transplanted Mice Develop Milder RR-EAE Than Their Untreated Counterparts

**Figure 1:**
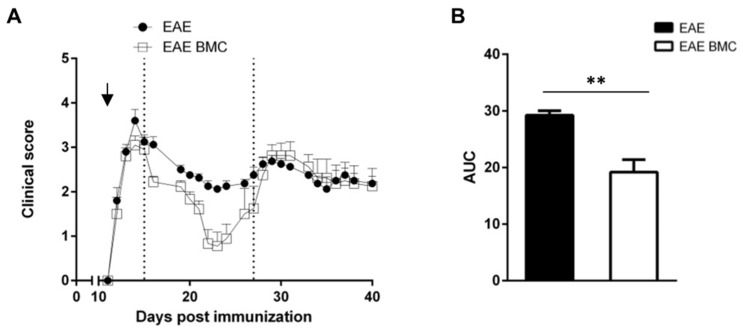
Mice treated with HSCs after intense immunosuppression develop milder EAE than their untreated counterparts only during the first relapsing-remitting phase. (**A**) EAE course of mice untreated or irradiated and treated with BMC. A representative example of two independent experiments is presented (total mice tested per group in two experiments n = 21). The arrow indicates the day when treatment was performed. Data are shown as mean ± SEM daily clinical score. (**B**) Quantification of the area under the curve (AUC) corresponding to the first relapse (days 15 to 27 after disease induction, as delineated by the two dotted lines on panel (**A**) during the course of the disease). Data are shown as mean ± SEM. ** *p* < 0.01.

We studied the effect of the intravenous administration of BMC after a lethal dose of γ radiation on the disease course of RR-EAE in SJL/J mice. All mice induced for RR-EAE showed clinical signs from 12 days post-immunization (dpi) with PLP139–151 (Figure 1a), together with the loss of body weight (data not shown). On the day of onset, mice were treated with intense immunosuppression, through TBI and BMC transplantation; control EAE-affected mice received the same volume of PBS. As can be seen in Figure 1a, the first acute disease phase occurred at the same time for both groups. Both treated (EAE BMC and EAE BMC MSC groups) and untreated mice (EAE group) exhibited a relapsing-remitting course of EAE, as expected, albeit with significantly greater motor recovery at the remission phase in mice treated with BMC. The assessment of clinical severity via the measurement of the area under the curve (AUC; Figure 1b) showed a significantly greater recovery from neurological impairment in mice treated with BMC in the remission stage, spanning days 15–27 after disease induction.

### 3.2. Co-Transplantation of MSCs with BMC Has No Synergistic Beneficial Effect on RR-EAE

**Figure 2:**
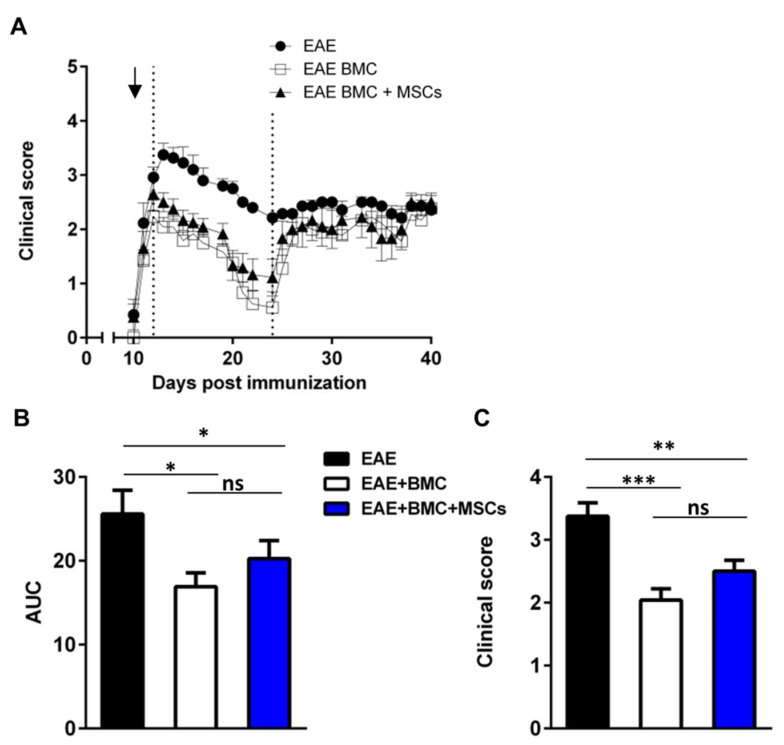
Co-transplantation of HSCs and MSCs has no synergistic beneficial effect on EAE course or severity. (**A**) EAE course of mice untreated, irradiated and treated with HSCs, or irradiated and co-transplanted with HSCs and MSCs (total mice tested per group n = 13). The arrow indicates the day when treatment was performed. Data are shown as mean ± SEM of daily clinical score. (**B**) Quantification of the area under the curve (AUC) corresponding to the first relapse (days 13 to 23 after disease induction, as delineated by the two doted lines on panel (**A**) during the course of the disease). Data are shown as mean ± SEM. * *p* < 0.05. (**C**) Comparison of the mean of clinical score between groups of mice untreated, irradiated and treated with HSCs, or irradiated and co-transplanted with HSCs and MSCs, at disease peak (day 14). ns refers to “not-statistically significant”. Data are shown as mean ± SEM. ** *p* < 0.01, *** *p* < 0.001.

Because MSCs constitute an essential BMC niche component and play an important role in the hematopoietic engraftment, we theorized that co-transplantation with BMC and MSCs in RR-EAE could promote hematopoietic engraftment and accelerate lymphocyte recovery. To address this question, we analyzed three groups of mice, all induced for RR-EAE and subjected to TBI: (a) untreated; (b) treated with BMC administration; and (c) treated by co-transplantation with MSCs plus BMC. The extent of recovery at the remission phase (between 13 and 23 dpi) was more pronounced than in untreated controls in all treated mice (Figure 2a) and there was no apparent difference in the RR-EAE clinical course between mice treated with BMC alone or co-transplanted with MSCs, as assessed by measurement of the AUC (Figure 2b). The analyses of clinical scores at the disease peak (14 dpi) revealed that the mean clinical scores of mice treated with BMC alone (2.041 ± 0.17) or co-transplanted (2.500 ± 0.17) were reduced in comparison with those of untreated mice (3.375 ± 0.21) (Figure 2c).

### 3.3. TBI Induces an Aplastic Phase in RR-EAE-Affected Mice That Is Reverted as a Result of BMC Administration; Co-Transplantation with MSCs Does Not Increase Immune Cell Engraftment

**Figure 3:**
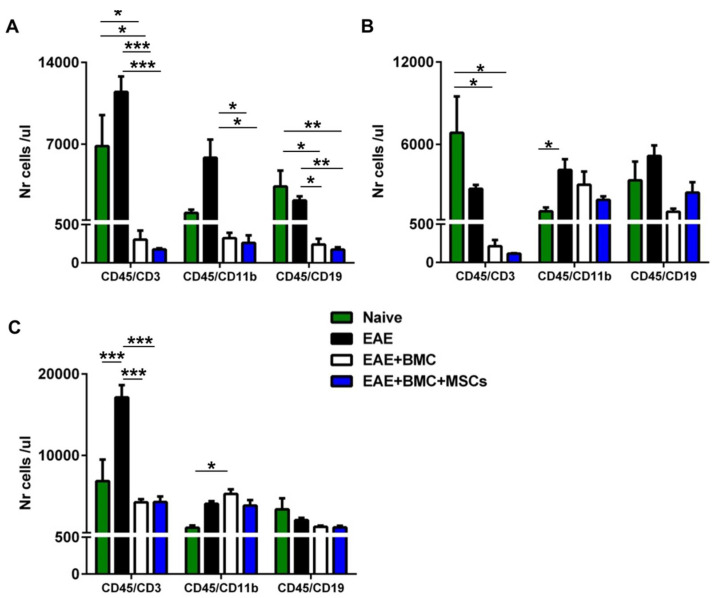
Injection of MSCs together with HSCs does not enhance immune cell engraftment. Quantification of immune cells performed by counting the absolute number/μL of EDTA-treated peripheral blood of T cells (CD3), monocyte/macrophages (CD11b) and B cells (CD19) at: (**A**) 3 days after TBI, (**B**) 12 days after TBI, and (**C**) 33 days after TBI. Quantitative data are presented as mean ± SEM of CD45-gated cells with n = 3 mice per group. * *p* < 0.05, ** *p* < 0.01, *** *p* < 0.001.

The timing of the reconstitution and regain of function of a donor-derived immune system is of importance for recovery and long-term survival after BMC transplantation. Accordingly, we sought to evaluate the extent and timing of hematopoietic engraftment and immune-reconstitution in RR-EAE-affected mice irradiated and treated with BMC alone or with BMC plus MSCs (co-transplantation) by counting the immune circulating cells, T cells, B cells and monocyte/macrophages, throughout the disease course. New blood cells usually take 10–14 days to reappear in the peripheral blood after TBI and the transplantation of BMC [16]. Accordingly, in order to verify the effect of TBI and monitor immune reconstitution, we quantified the cells on days 3, 12 and 33 after treatment. Flow cytometry experiments revealed that, following TBI, the numbers of circulating immune cells, CD3+ T cells, CD11b+ monocyte/macrophages and CD19+ B cells in treated mice were significantly decreased 3 days after treatment, as expected, compared to their naïve counterparts or to untreated RR-EAE-affected mice (Figure 3a). At 12 days post-treatment, the numbers of circulating CD3+ T cells were still reduced to the same extent in RR-EAE-affected mice treated with BMC alone or with BMC and MSCs. At the same time point, the numbers of CD11b+ monocyte/macrophages and CD19+ B cells were not statistically different between the groups treated with BMC and MSCs, and had increased to numbers comparable to those of naïve mice (Figure 3b). At 33 days after treatment, the number of circulating T cells in untreated RR-EAE-affected mice had increased in comparison with naïve mice and with the groups treated with BMC and MSCs. In contrast, the number of circulating T cells in the groups treated with BMC and MSCs, although still reduced, had reverted to levels comparable to those of naïve mice. In mice treated with BMC alone the number of circulating monocyte/macrophages was only increased as compared to naïve mice. Circulating B-cell numbers did not differ among all the groups (Figure 3c).

### 3.4. The Plasma Level of IL4 Is Significantly Elevated in Co-Transplanted Mice

**Figure 4:**
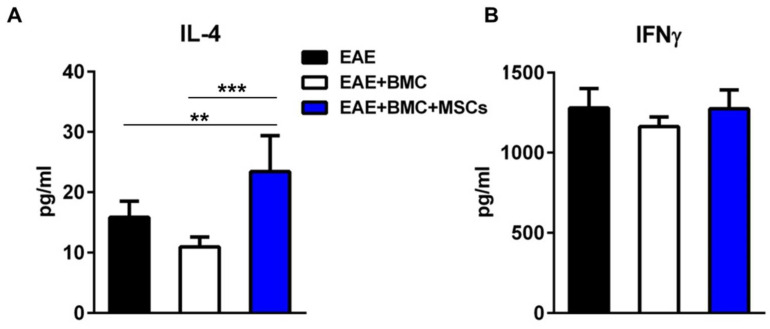
Anti-inflammatory IL-4 is upregulated in co-transplanted mice. Quantification of (**A**) IL-4 and (**B**) IFN-γ in plasma from EAE-affected mice untreated, irradiated and treated with HSCs, or irradiated and co-transplanted with HSCs and MSCs, at 12 days post-treatment. Data are presented as mean ± SEM cytokine concentration of triplicate plasma samples of n = 4 mice per group. ** *p* < 0.01 *** *p* < 0.001.

The injection of MSCs into EAE-affected mice mediates immunomodulatory effects observed ex vivo, resulting in the suppression of T-cell proliferation and in the shift from pro-inflammatory Th1 to anti-inflammatory Th2 cells, inducing a change in the cytokine profile towards anti-inflammation [7,12,17]. Accordingly, we compared the levels of IFNγ and IL-4 in the plasma of mice treated with BMC alone or co-transplanted with MSCs, at the initiation of the remitting phase, that is, at 12 days post-treatment. As shown in Figure 4a, the plasma levels of IFN-γ were unchanged by the treatment of EAE-affected mice with BMC alone or together with MSCs. In contrast, the levels of IL-4 upon co-transplantation were significantly upregulated as compared to those of RR-EAE-affected mice untreated or treated with BMC alone (Figure 4b). These data suggest that MSCs might play a role as an anti-inflammatory partner in the co-transplantation.

## 4. Discussion

In this work, we have shown that RR-EAE-affected mice transplanted with syngeneic BMC, or with a combination of HSC-containing syngeneic BMC and MSCs, following intense immunosuppression with TBI, undergo a disease course with clinical signs at the remission phase that are considerably lower than those observed in untreated mice. Although our hypothesis was that co-transplantation with BMC and MSCs would synergize the therapeutic effect of each treatment observed in this and previous studies, we did not observe any enhancement of engraftment or increased rapidity of reconstitution upon co-transplantation, nor did co-transplantation lead to an amelioration of the disease course, as compared to treatment with BMC alone. Damage of the target tissues in MS and its animal model EAE, i.e., in the brain and spinal cord, is most likely mediated by immune effector cells such as T and B cells and monocyte/macrophages [18]. Targeting the immune system with conventional therapeutic approaches based on immunosuppressant drugs and immunomodulatory agents has modified the management of the relapsing-remitting (RR) form of MS, resulting in the amelioration of the clinical course and slowing down deterioration [19]. In contrast, the conventional immunosuppressive and immunomodulatory therapeutic approach is inefficient in rapidly evolving patients and in severe forms of RR-MS [20]. Intense immunosuppression followed by autologous HSC infusion is a therapeutic strategy that is currently utilized in rapidly evolving aggressive forms of RR-MS that are unresponsive to the available treatments, with clinical and magnetic resonance imaging signs of active disease, when the severity of the disease course outweighs the acute toxicity and the transplant-related mortality risk of the procedure (although this risk is decreasing, and was less than 1% for those carried out in the years between 2012 and 2016) [3,21]. Mortality is usually related to the risk of engraftment failure concomitantly with that of infection during the neutropenic period that follows the conditioning regimen [4]. Although this is the first report showing the effect of immunosuppression followed by BMC transplantation for RR-EAE induced in SJL/J mice by immunization with PLP139–159, this procedure has been shown to prevent and/or reduce the clinical signs of acute monophasic EAE induced in rats by immunization with a CNS homogenate as an encephalitogen [11,22,23,24]. The mechanisms underlying the protective effects of this therapeutic approach are believed to be mainly due to the establishment of antigen-specific tolerance, presumably through a mechanism of clonal deletion or anergy [25]. Indeed, autoreactive lymphocytes, which retain the potential to induce CNS autoimmunity, are part of the normal lymphocyte repertoire, and perturbation of the clonal selection, such as that which occurs in EAE, may be in part responsible for the relapsing-remitting forms of the disease in both humans and mice [26,27]. In RR-EAE, T cells are repeatedly recruited from the periphery towards neuroantigens, and through epitope spreading [28,29,30] towards neo-antigens, emerging as a result of tissue damage, thereby reinforcing the local inflammatory reaction within the CNS and leading to clinical relapses. In the human disease, the protective effect of HSC transplantation leads to the long-term suppression of the inflammatory activity in patients and the efficacy does not depend on persisting lymphopenia but is associated with profound qualitative immunological de novo regeneration of the T-cell compartment [31]. In the present work, we showed that BMC transplantation enhanced disease amelioration in the first remission phase of EAE, suggesting that the therapy had a short-term impact. We suggest that, later on, the beneficial effect of the treatment is overcome by the local reinforced immune cell reactivation in the periphery. Possible explanations for the lack of beneficial synergy of co-transplantation may be related to the high inflammatory environment at the acute phase of the disease or to the status of the transplanted BMC themselves. BMC require in vitro exposure to a combination of growth factors and cytokines for optimal proliferation and differentiation in vivo [32]. In this context, the limited effects of BMC treatment in EAE-affected mice might be attributable to the absence of pre-conditioning. To overcome this problem in the present work, we had sought to enhance the capacity of BMC to proliferate, differentiate and possibly engraft by administering MSCs concomitantly. Per se, MSCs play an immunomodulatory role in RR-EAE when administered at disease onset and at the peak of the disease, decreasing inflammatory infiltrates and demyelination [12]. MSCs are prone to induce peripheral tolerance, as demonstrated by the inability of T cells isolated from lymph nodes of EAE-affected MSC-treated mice to proliferate when re-stimulated with the encephalitogen [12]. Due to their physiological trophic role, MSCs have been characterized by their ability to promote engraftment in transplantation [33] and have been demonstrated to promote hematopoietic engraftment and to accelerate neutrophil recovery when injected together with HSCs in humans [34]. Numerous studies have reported that, in the transplantation of autologous HSCs, co-transplantation of expanded MSCs reduces the risk of graft failure, accelerates BMC proliferation, inducing a rapid hematopoietic recovery [35] and, consequently, engraftment [14]. The quantification of engraftment in the majority of the studies has been addressed by counting the absolute number of neutrophils. Since the nature of the role of neutrophils in inflammation in EAE pathogenesis is still debated and since we hypothesized that the co-transplantation might be involved in the establishment of T-cell tolerance, we quantified the number of EAE-relevant immune cell types that play a fundamental role in the immune response in EAE, such as T cells, B cells and monocytes/macrophages. In our hands, MSCs co-transplanted with BMC did not enhance the presence of circulating immune cells at the different post-treatment time points considered and thus, we suggest that MSCs do not accelerate immune reconstitution when administered together with BMC. In line with our data, Lazarus et al. showed that the administration of culture-expanded allogeneic MSCs four hours before the infusion of HSCs in 46 patients undergoing myeloablative HSC transplantation did not accelerate hematopoietic engraftment [15]. It is not clear why MSCs apparently do not support BMC engraftment and faster immune reconstitution because phylogenetically they play a trophic role for those cells; we can only speculate that the long-term in vitro culturing of MSCs affects their physiological function. There is strong preclinical evidence from our and other laboratories of the robust immunomodulatory properties of MSCs per se when used as therapeutic agents in EAE-affected mice. In fact, MSCs mediate immunomodulation through a synergy of cell contact-dependent mechanisms and soluble factors, reducing the activation of pro-inflammatory dendritic cells and promoting IL-4-producing T cells (Th2 and Tregs) [36,37]. Because MSC transplantation is associated with anti-inflammatory effects [12], we assessed the circulating IL-4 levels in the presence or absence of MSCs in the treatment and found increased levels of IL-4 in RR-EAE-affected co-transplanted mice as compared to mice treated with BMC alone. Notably, the increased circulating amount of IL-4 in co-transplanted EAE-affected mice was not accompanied by an amelioration in terms of clinical EAE severity. Similar observations were made in other studies, where the protective role of IL-4 during EAE failed to be completely explained by the polarization of the Th1 immune responses towards IFN-γ–reduced Th2 immune responses [38,39,40]. Thus, although IL-4 might be an important Th2 cytokine involved in immunomodulatory processes in EAE, the mode of action by which it suppresses the encephalitogenic response remains enigmatic. As the increase in IL-4 level is apparently related to co-transplantation with MSCs, it is possible that multiple injections of these cells would result in the maintenance of elevated IL-4 levels, promoting a shift to an anti-inflammatory environment, with an ensuing effect on disease expression.

## 5. Conclusions

We showed that the infusion of BMC after TBI impacts RR-EAE at the remission of the acute phase, with no synergistic effect of co-transplantation with MSCs. Our data in RR-EAE-affected mice further support the suggestion that co-transplantation with MSCs does not sustain the hematopoietic engraftment after BMC transplantation.

## Data Availability

The data that support the findings of this study are available from the corresponding author upon reasonable request.
